# Single Residues in the Outer Pore of TRPV1 and TRPV3 Have Temperature-Dependent Conformations

**DOI:** 10.1371/journal.pone.0059593

**Published:** 2013-03-26

**Authors:** Sung Eun Kim, Ardem Patapoutian, Jörg Grandl

**Affiliations:** 1 Department of Molecular and Cellular Neuroscience, Dorris Neuroscience Center, The Scripps Research Institute (TSRI), La Jolla, California, United States of America; 2 Genomic Institute of the Novartis Research Foundation (GNF), San Diego, California, United States of America; Duke University Medical Center, United States of America

## Abstract

Thermosensation is mediated by ion channels that are highly temperature-sensitive. Several members of the family of transient receptor potential (TRP) ion channels are activated by cold or hot temperatures and have been shown to function as temperature sensors in vivo. The molecular mechanism of temperature-sensitivity of these ion channels is not understood. A number of domains or even single amino acids that regulate temperature-sensitivity have been identified in several TRP channels. However, it is unclear what precise conformational changes occur upon temperature activation. Here, we used the cysteine accessibility method to probe temperature-dependent conformations of single amino acids in TRP channels. We screened over 50 amino acids in the predicted outer pore domains of the heat-activated ion channels TRPV1 and TRPV3. In both ion channels we found residues that have temperature-dependent accessibilities to the extracellular solvent. The identified residues are located within the second predicted extracellular pore loop. These residues are identical or proximal to residues that were shown to be specifically required for temperature-activation, but not chemical activation. Our data precisely locate conformational changes upon temperature-activation within the outer pore domain. Collectively, this suggests that these specific residues and the second predicted pore loop in general are crucial for the temperature-activation mechanism of these heat-activated thermoTRPs.

## Introduction

**Figure 1:**
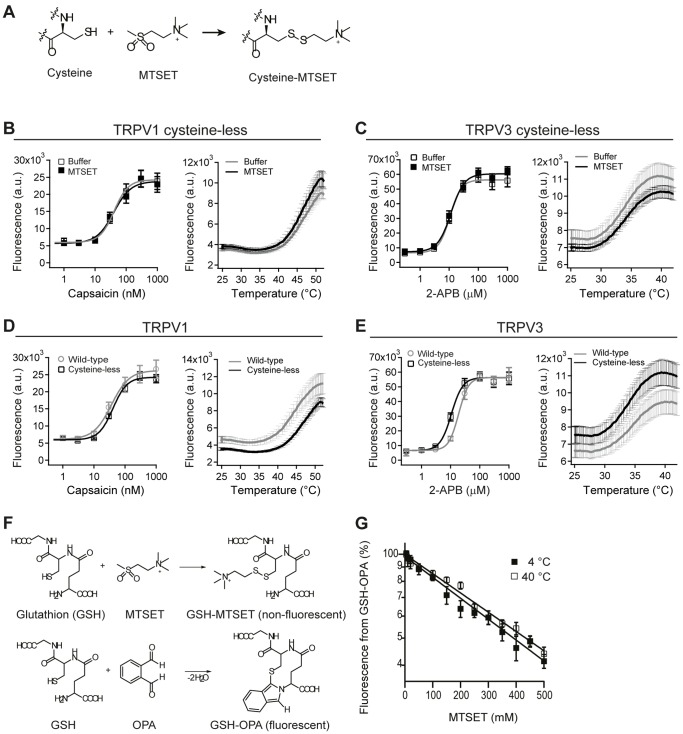
Cysteine scanning on TRPV1 and TRPV3 channels. (A) Schematic of MTSET labeling on a cysteine residue by covalent bond formation. (B–D) Chemical- and temperature- dose-response curves of cysteine-less TRPV1 after application of MTSET or buffer control (B), of cysteine-less TRPV3 after application of MTSET or buffer control (C), of cysteine-less and wild-type TRPV1 (D), and of cysteine-less and wild-type TRPV3 (E). For chemical dose-responses data are averages of five measurements (mean ± s.d.) and for temperature-responses averages of 16 wells (mean ±2× s.e.). (F) Reaction schemes of GSH with MTSET and GSH with OPA. (G) Fluorescence signal of obtained from competing reactions in (F) as a function of MTSET concentration at 4°C and 40°C. n>20 with from three independent experiments. Error bars are mean ± s.d.

Thermosensation is mediated by sensory neurons that are uniquely excitable by distinct temperatures. Some neurons respond to innocuous hot or cold temperatures, while nociceptive neurons respond to both noxious hot and cold temperatures that are perceived as painful. The conversion of thermal stimuli into neuronal activity is at least partly carried out by a subset of transient receptor potential ion channels (called thermoTRPs) [1] [2]. Expression of these receptors is sufficient to make a naïve cell responsive to heat or cold and genetic ablation of distinct thermoTRPs in mice has been shown to cause specific loss of neuronal excitability by temperatures, altered behavioral temperature-sensitivity and thermal nociception [3] [4] [5]. ThermoTRPs are multimodal receptors that are activated by temperatures with distinct thresholds and a variety of physically distinct stimuli such as chemicals, voltage and pH [2] [6] [7]. Whereas the mechanisms of ion channel gating by chemicals and voltage are understood in principal, the logic of temperature-activation remains unknown. An important first step towards this mechanism would be the identification of structures (residues) involved in temperature-activation. This structural information is now starting to emerge from studies using chimeric or unbiased mutagenesis screens [8] [9] [10] [11] [12] [13] [14]. Previously, two studies from our lab showed that single point mutations in TRPV3 and TRPV1 can specifically affect temperature-activation without altering responses to chemical agonists [8] [9]. The identified mutations are not randomly distributed throughout the protein, but cluster in the pore domain, suggesting a mechanistic involvement of the pore domain in temperature-activation. In a complementary approach Yang and colleagues used fluorescence resonance energy transfer (FRET) and detected conformational changes across subunits of the outer pore domain upon temperature-activation, but not chemical- or voltage-activation [15]. However, both results are not strict proof that during temperature-activation conformational changes occur within the pore domain: for example, point mutations might act allosterically on structures outside the pore and thus affect the temperature-gating transition. In addition, FRET is only sensitive to global conformational changes, but gives little information about localized structural changes. Certain structural and functional aspects of the TRPV1 and TRPV3 pore-domains have already been addressed by previous studies [16] [17] [18]. However, much less is known about how specifically temperature affects the pore structure. In order to identify small structural changes upon temperature-activation with single amino acid resolution, we applied the substituted cysteine accessibility method (SCAM) to TRPV1 and TRPV3. Cysteine accessibility has been widely used to determine conformational changes of single residues in many ion channels [19]. In brief, solvent-accessible cysteine residues are chemically labeled by reactive compounds such as methanethiosulfonate (MTS). This chemical labeling may induce changes in ion channel functions, which can be measured. Here, we used 2-(trimethylammonium) ethyl methanethiosulfonate (MTSET) as a chemical label, because this positively charged MTS reagent is membrane-impermeable and thus suitable to specifically probe extracellular structures of transmembrane proteins (Figure 1A). We screened 22 and 30 residues within the outer pore-region of TRPV1 and TRPV3 respectively and compared solvent-accessibility to MTSET at high or low temperatures. We identified single residues with temperature-dependent accessibilities for extracellular solvent in both heat-activated TRP channels.

## Results

### Temperature-dependent Cysteine-accessibility Assay for TRPV1 and TRPV3

In order to assure site-specificity of MTSET labeling, we constructed rat TRPV1 and mouse TRPV3 channels lacking endogenous cysteines with potential accessibility to the extracellular solvent. For TRPV1 we engineered mutations C616W, C621S and C634S and for TRPV3 mutations C550F, C612A and C619A. When tested for their responsiveness to chemical agonists or temperature, both extracellular cysteine-less (cysteine-less for short) channels were inert to previous application of 10 mM MTSET (Figure 1 B, C). Moreover, we found that both cysteine-less channels responded similarly to chemical agonists or temperature, when compared to the respective wild-type channels (Figure 1 D, E). Next, we selected candidate residues for the cysteine-accessibility screen. Using homology models of TRPV1 and TRPV3 that are both based on the crystal structure of Kv1.2 (44% homology for the pore region of TRPV1 and 54% for TRPV3) [8] [9], we predicted residues to be in proximity to amino acids that we previously identified to be specifically required for temperature activation [8] [9]. For TRPV1 we selected 22 and for TRPV3 30 residues, all located within the pore-domains (Figure S1). Next, we substituted each selected residue individually into a cysteine in the background of the respective cysteine-less channel. In order to probe the accessibility of single introduced cysteines in a temperature-dependent manner, we incubated HEK293 cells transiently expressing mutant channels or cysteine-less controls with MTSET or buffer control at cold (20±2°C) or hot (42±2°C) temperatures (see Methods for details). We then tested the activity of these channels in response to thermal-activation in a Fluo-3 calcium-influx assay. All experiments were performed in a 384-well plate format. This allowed us to simultaneously test and compare several cysteine mutant constructs and cysteine-less controls with MTSET labeling or buffer control within a single experiment.

To assure that our assay measures temperature-dependent accessibilities of a cysteine residue rather than the temperature-dependent reactivity of MTSET, we measured reaction kinetics of MTSET with cysteine residues in solution at 4°C and 40°C. As a source of cysteine residues, glutathione (GSH), a cysteine-containing tri-peptide (glutamic acid-cysteine-glycine) was incubated with various concentrations of MTSET. The amount of non-reacting GSH was probed in a competing reaction by ortho-phthalaldehyde (OPA). OPA reacts with GSH to yield the fluorescent product GSH-OPA, which we measured with a fluorescent plate reader (Figure 1F). Our results show that the reaction rate of MTSET at 4°C (518±18 M^−1^·s^−1^) is not substantially different at 40°C (477±21 M^−1^·s^−1^) (Figure 1G). We therefore conclude that any substantial changes in channel function we measure in our assay reflect changes of solvent accessibility to MTSET.

### Temperature-dependent Solvent-accessibility Changes in TRPV1

**Figure 2:**
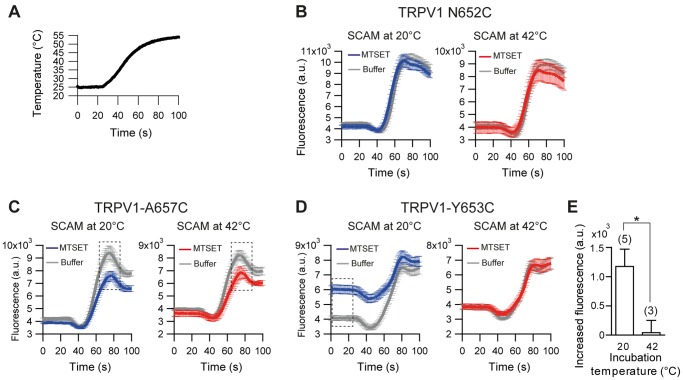
Temperature-dependent labeling in TRPV1. (A) Temperature as a function of time during FLIPR temperature-activation assay. (B–D) Representative examples of fluorescence responses upon temperature stimulation of TRPV1 N652C (B), A657C (C) and Y53C (D) after incubation of MTSET at 20°C (blue) and 42°C (red). For both temperatures, buffer as a negative control is colored gray; n>8 wells. Error bars are 2× s.e. (E) Average basal fluorescence change of TRPV1 Y653C after incubation of MTSET at 20°C and 42°C. The basal fluorescence is averaged fluorescence level between 0 to 20 sec. For each experiment, the increase of basal fluorescence was obtained by subtracting buffer control from MTSET incubation. Five independent experiments were performed for 20°C and three for 40°C with n>5 wells per experiment. Two-tailed t-test, *p = 0.036. Error bars are mean ± s.e.

In TRPV1 we screened 22 residues for their accessibility to MTSET either at 20°C or 42°C. We selected these two temperatures, because at 20°C wild-type TRPV1 is predominantly in the closed state, whereas 42°C induces channel opening [7]. For 20 residues we did not observe any changes in channel function in response to thermal-activation upon application of 2 mM MTSET at either temperature (Figure 2A, B and data not shown). We conclude that these residues are either not accessible to the extracellular solvent or that chemical modification by MTSET does not perturb temperature-activation of TRPV1 at these locations.

In contrast, labeling of residue A657C resulted in altered channel function at both temperatures to a similar extend: upon MTSET incubation maximal temperature-induced responses were similarly decreased compared to buffer control (28.8±2.5% at 20°C vs. 30.1±5.5% at 42°C) (Figure 2C). This result suggests that residue A657C is exposed to the extracellular solvent and that the accessibility is not strongly temperature-dependent. Alternatively, it is possible that the conditions under which we performed MTSET labeling were saturating and therefore differences in solvent accessibility were not detected.

Interestingly, for residue Y653C we discovered that channel function depended strongly on the labeling temperature. At 20°C MTSET incubation resulted in a strong increase of the basal fluorescence signal (48.1±7.2% compared to buffer control). However, when cells were incubated with MTSET at 42°C, the basal fluorescence was not affected (0.0±5.4% compared to buffer control) (Figure 2D, E). This result indicates that residue Y653C is accessible to the extracellular solvent at 20°C, but not at 42°C. Therefore, we were able to identify a residue in the pore domain of TRPV1, which has temperature-dependent solvent accessibility.

Residue Y653 is in close proximity to E648, which has been shown to mediate sensitivity to low pH [20]. To understand if residue Y653 is also important for acid-activation, we measured its pH dose-responses (Figure S2). Our data show that the Y653C mutant channel is activated by extracellular low pH, but that sensitivity and efficacy are reduced compared to cysteine-less or wild-type channels (EC_50_: Y653C: 6.2±0.1; cysteine-less: 6.4±0.1; wild-type: 6.4±0.1, normalized maximum responses: Y653C: 17.6±1.5%; cysteine-less: 66.8±2.8%; wild-type: 100±2.2%). The shift in the EC_50_ is reminiscent of our previous report where the tyrosine residue was mutated into threonine (Y653T: 6.2±0.3 and wild-type: 6.7±0.3) [9]. However, Y653T had normal maximum responses when compared to wild-type TRPV1 (Y653T: 132±10.9%; wild-type: 100±10.2%). These experiments demonstrate that depending on the side-chain residue Y653 can affect the acid-activation of TRPV1 either directly or indirectly.

### Temperature-dependent Solvent-accessibility Changes in TRPV3

TRPV3 is like TRPV1 a heat-activated TRP channel, but with a lower temperature-threshold (33–39°C) at physiological levels [1] [2]. Overall, TRPV3 is predicted to have a pore-domain similar to TRPV1 (61.8% homology). However, the predicted extracellular domains are distinct in both proteins. In TRPV3, the first predicted extracellular loop is 15 amino acids shorter compared to TRPV1 and the second predicted extracellular loop has poor homology. Nevertheless, in TRPV3 single point-mutations that specifically affect temperature-, but not chemical-activation are located in the pore-domain as well [8]. Despite little sequence homology, functional configurations important for temperature-gating might therefore be conserved. To test this idea we applied the same cysteine-scanning assay to TRPV3. Similarly to TRPV1, we selected thirty candidate residues based on a TRPV3 homology model [8], engineered single-cysteine mutants, subjected them to MTSET incubation at different temperatures and tested their function.

Of all 30 residues that we tested 24 did not show significant changes in channel function upon MTSET incubation (data not shown). As mentioned above, it is likely that these residues are either inaccessible to MTSET or that chemical modification does not affect channel function. However, at four residues (I609C, K611C, S626C and L653C), channel function was similarly altered upon MTSET treatment at 20°C and 40°C (Figure S3). These data suggest that these residues are accessible to the extracellular solvent independently of temperature, or it is possible that differences are not detected if the MTSET concentration was saturating.

**Figure 3:**
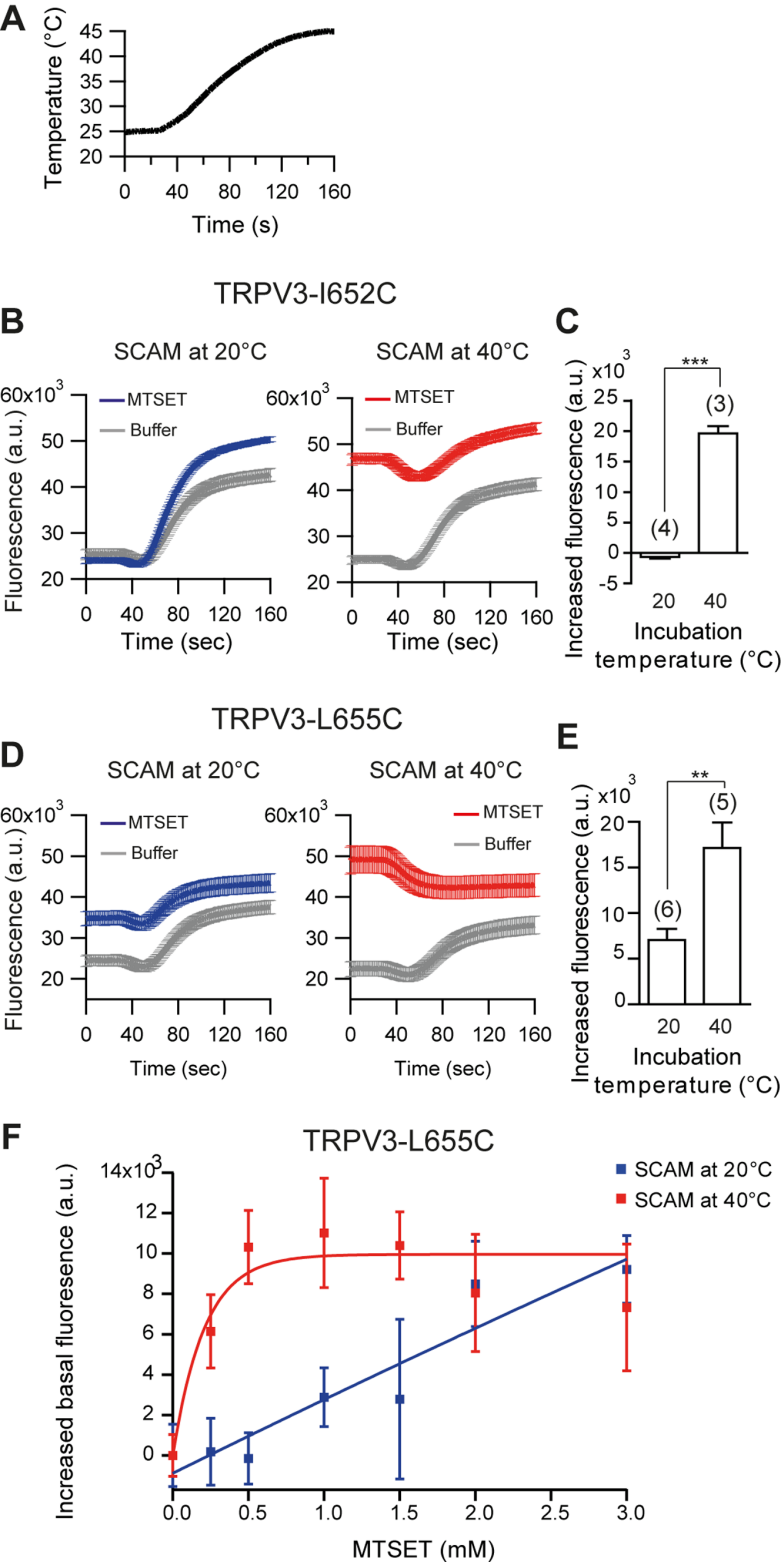
Temperature-dependent labeling in TRPV3. (A) Temperature as a function of time during FLIPR temperature-activation assay. (B) Representative examples of fluorescence responses upon temperature stimulation of TRPV3 I652C. n>8 wells. Error bars are mean ±2x s.e. (C) Average basal fluorescence change of TRPV3 I652C after incubation of MTSET at 20°C and 40°C. For each experiment, the basal fluorescence is an average of fluorescence between 0 and 20 sec. Fluorescence change is the difference of MTSET incubation and buffer incubation basal fluorescence. Numbers of independent experiments are shown in the bar graph and n>8 wells per experiment. Error bars are mean ± s.e. Two-tailed t-test, ***p<0.0001. (D) Representative examples of fluorescence responses upon temperature stimulation of L655, n>8 wells, Error bars are mean ±2× s.e. (E) Average basal fluorescence change of TRPV3 L655C after incubation of MTSET at 20°C and 40°C. Number of experiments is shown in the bar graph and n>8 wells per experiment. Error bars are mean ± s.e. Two-tailed t-test, **p = 0.0062. (F) The basal fluorescence change of TRPV3 L655C after incubation of MTSET at 20°C and 40°C as a function of MTSET concentration. The incubation time was 10 min. n>5 wells. Error bars are mean ± s.d. Straight lines are exponential fits to the data.

At two positions, I652C and L655C, we observed that MTSET labeling affected channel function in a temperature-dependent manner. At residue I652C, MTSET incubation at 40°C caused a strong increase in basal calcium levels (88.5±4.6%), whereas incubation at 20°C had no substantial effect (−5.2±1.0%). At residue L655C MTSET incubation at 20°C resulted in elevated basal calcium levels compared to incubation with buffer control (42.2±4.9%). Moreover, this effect was substantially stronger at 40°C (118.5±12.3%) (Figure 3A–E). To better quantify the temperature-dependence for residue L655C we measured changes in basal calcium levels as a function of the MTSET concentration (Figure 3F). When fitting these data with a first order reaction kinetics we find that the rate of cysteine modification is 31.366±432 mM at 20°C and 0.208±0.112 mM at 40°C. Overall, this observation is consistent with residues I652C and L655C being partially exposed to the extracellular solvent at 20°C and to a greater extent at 40°C. In summary, we found strong evidence that single residues in the outer pore domain of TRPV1 and TRPV3 undergo temperature-dependent conformational changes.

### Electrophysiological Measurement of Solvent-accessible Changes in TRPV1 Y653C

We wanted to confirm our results from calcium-influx screen with electrophysiological recordings of channel currents for the cysteine mutants. Since TRPV3 is strongly sensitized by repeated stimulations [8], we reasoned that the best suited residue for this would be TRPV1 Y653C.

**Figure 4:**
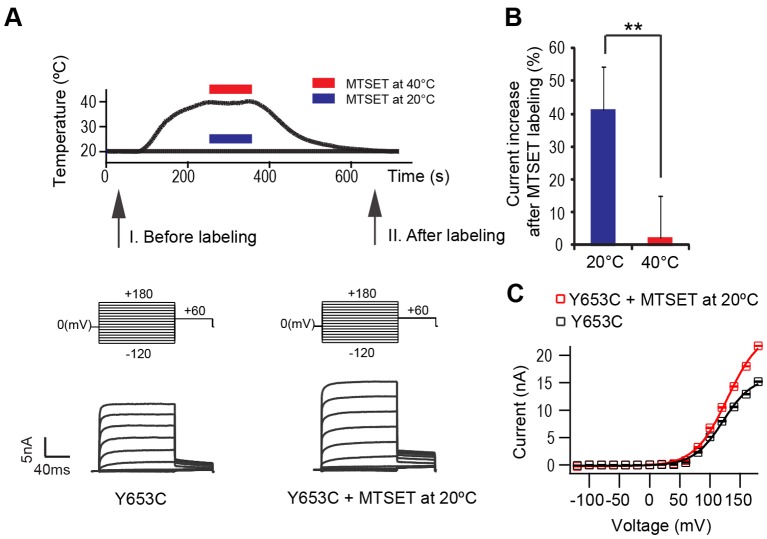
Electrophysiological characterization of temperature-dependent MTSET accessibility of TRPV1 Y653C. (A) Above: Temperature profile for a whole-cell voltage-step protocol. Middle: Voltage-step protocols were applied before (I) and after (II) MTSET application at 20°C or 40°C. Bottom: Example of current traces of Y653C before and after 2 mM MTSET at 20°C. (B) Average change of plateau current (+100 mV) upon application of MTSET at 20°C or 40°C. Data are averages from five patches. Two-tailed t-test, **p = 0.00034. (C) Current-voltage (IV) curves from whole-cell measurement. Error bars are mean ± s.e.

We therefore performed whole-cell patch-clamp experiments with HEK293 cells, expressing TRPV1 Y653C channels. To test modified channel-activation by MTSET labeling, we applied two voltage-step protocols: one before and the other after application of MTSET (2 mM, 100 s). To test temperature-dependency of the modifications, the MTSET was applied either at low (20°C) or high (40°C) temperatures. Consistent with the previous FLIPR result, we observed after MTSET application at low temperatures (20°C) an increase of 41±13% (n = 15 from 5 cells) in the plateau-current evoked at a holding potential of +100 mV, whereas MTSET (2 mM, 100 s) application during high temperatures (>40°C) failed to cause any substantial current increase (2±13%, n = 15 from 5 cells) (Figure 4). This confirms our observation that residue Y653C is preferentially labeled at cold, but less at hot temperatures. Thus, the temperature-dependent conformational change of TRPV1 Y653C is strongly supported by two independent approaches.

## Discussion

The temperature-activation mechanism of TRP ion channels is not well understood. Although structures that regulate this activation have started to emerge, the detailed conformational changes involved in the gating transition are unknown. Here, we aimed to identify conformational changes induced by temperature-activation of heat-sensitive thermoTRP channels. We focused our study on the outer pore-domains of TRPV1 and TRPV3, because previous studies pointed towards conformational changes in these structures [8] [9]. Nevertheless, within these structures we used an unbiased approach to ask if and where such changes might occur. With a combination of cysteine-accessibility experiments and a high-throughput functional assay we were able to efficiently screen a total of 52 candidate residues at two distinct temperatures. Cysteine-accessibility has been applied to study conformational changes of many ion channels, including TRPV1. Salazar and colleagues screened residues in transmembrane domain 6 of TRPV1 and identified I668C and Y671C to have both temperature- and capsaicin-dependent accessibilities toward silver ions from the intracellular side [21].

**Figure 5:**
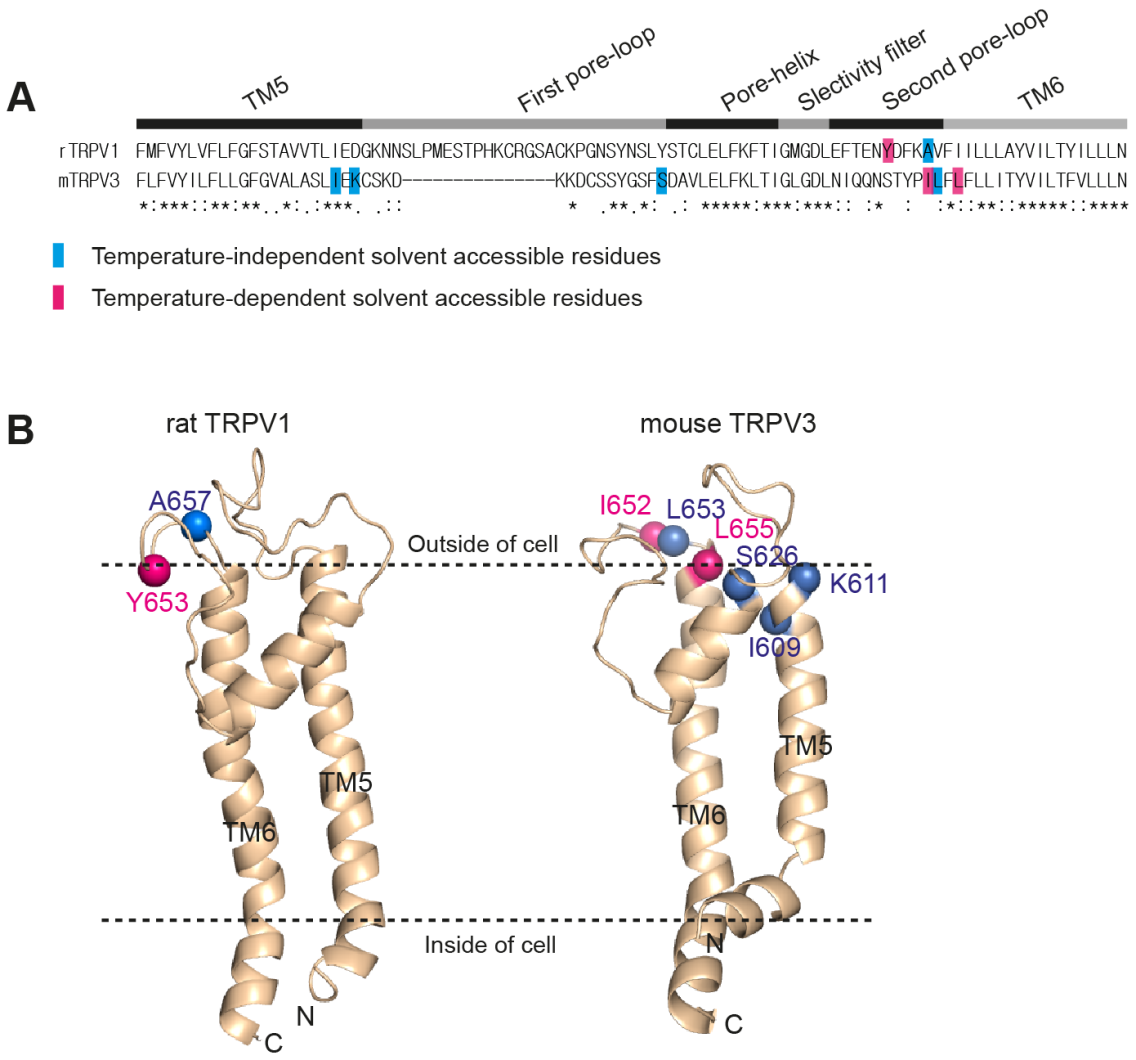
Location of temperature-dependent accessible residues. (A) Sequence alignment of pore domains of rat TRPV1 and mouse TRPV3. Predicted structural domains are indicated above. Residues with temperature-independent accessibility are highlighted in cyan and residues with temperature-dependent accessibility are in pink. (B) Homology models of pore domains. Same color coding was used as the above.

For TRPV1 the only residue we identified as temperature-specific is Y653C. Interestingly, this is one of the three residues that we had previously identified in an unbiased screen of 8,000 random mutant clones [9]. Mutation of this tyrosine residue into threonine reduces temperature-sensitivity and increases the apparent temperature-threshold [9]. Our data support the idea that this residue has a crucial role in temperature-gating and they suggest that mutations affect temperature-sensitivity by altering the related gating transition. However, the two other residues that can specifically affect temperature-activation upon being mutated (N628 and N652) were also tested in our screen, but did not show temperature-dependent accessibilities. This might indicate that mutations at these residues are not directly participating in temperature-gating, or alternatively that these residues are not accessible to the solvent. At this point we cannot exclude any of these distinct possibilities, but cysteine-scanning with other reactive compounds of different size or hydrophobicity might provide an answer. In TRPV3, residues I652C and L655C had temperature-dependent accessibilities. These residues are proximal but not identical to residues that can specifically alter temperature-activation upon being mutated [8]. For both ion channels we screened residues near the pore-domain (Figure 5A). Within this unbiased search, all residues we identified as temperature-dependent are located in the second predicted extracellular loop (Figure 5B). This is noteworthy, since there is active discussion over whether the first predicted extracellular pore-loop contributes to temperature-sensitivity [15] [22] [23].

Overall, it is surprising that we identified only a few, single temperature-dependent accessibility changes. This might suggest that the conformational changes involved in temperature activation are small and localized. Indeed, a recently proposed mechanism of temperature-activation suggests that conformational changes do not have to involve large, coherent domains [24]. More specifically, residues that experience hydrophobic changes in their environment are proposed to be the actual origin of temperature-sensitivity. Strikingly, all residues we identified are hydrophobic (tyrosine, isoleucine and leucine). However, the hydrophobic changes that these residues experience upon temperature-changes are opposite. In TRPV1 Y653C is exposed to the hydrophilic solvent at cold temperature, whereas in TRPV3 I652C and L655C experience more hydrophilic exposure at hot temperature.

Whether the pore-loops of TRPV1 and TRPV3 undergo very distinct conformational changes upon temperature-activation or if they are structurally identical is unclear for several reasons: First, the second extracellular pore-loop has poor sequence homology between TRPV1 and TRPV3 (36.3%), which makes a direct comparison of residue Y653 of TRPV1 with residues I652 or L655 in TRPV3 difficult. Second, the local solvent accessibility change of single residues may not be representative of the conformational change of the entire loop. Third, the conformational changes in the pore domain are more complex than described by a simple open-close mechanism [25]: For example, TRPV1 is desensitized and TRPV3 is sensitized by repeated stimulations [3] [26] [27] [28]. Also, the pore of TRPV1 is dilated by prolonged exposure to chemicals and protein-kinase C activation [17] and TRPV3 adapts to at least two distinct open states which elicit biphasic currents [16]. Moreover, the pore-domain of TRPV1 is known to confer sensitivity to extracellular acid. Mutational analysis showed that residues E600 and E648 of rat TRPV1 [20] and F660 of human TRPV1 [29] are critical for activation and potentiation by extracellular acid. Finally, our data show that residue Y653C is involved in both temperature and acid-activation (Figure S2 and [9]). Clearly, our experimental conditions do not capture all of these complex time-dependent and agonist-dependent mechanisms.

It is also unclear, if these residues undergo conformational changes along with other functionally more important domains, or if these residues are actual drivers of temperature-gating. In other words, we cannot say if these residues are ‘sensing’ temperature or translating the signal from another domain to the gate. A look at other proteins shows that loop structures might be active elements of temperature-sensitivity: For example, thermally-sensitive loops have been identified in glutamate and fructose-1,6- bisphosphatase [30] [31]. Furthermore, temperature-gated channels have been artificially engineered by introducing elastine-like polypeptites into the lumen of α-hemolysin [32]. In brief, our data give only an incomplete glimpse on the conformational changes of small domains within these large proteins. However, detecting conformational changes is the next logical step for a more complete understanding of the temperature-activation mechanism.

## Materials and Methods

### Mutagenesis

Cysteine-less and cysteine-substituents of TRPV1 or TRPV3 were generated from wild-type rat TRPV1 in pcDNA3.1 [9] or mouse TRPV3.1 in pcDNA3 [8] using the QuikChange II XL Site-directed Mutagenesis Kit (Stratagene). Constructs were fully sequence verified and DNA was prepared using Hi-Speed Plasmid Maxi Kit (Qiagen).

### Cell-culture/Transfection

HEK293 cells were maintained with Dulbecco’s minimal essential medium containing 4.5 mg ml−1 glucose, 10% heat-inactivated fetal bovine serum (vol/vol), 50 units/ml penicillin and 50 µg/ml streptomycin and incubated at 37°C with 5% CO_2_. Before transfection, the trypsinized HEK293 cells were pre-plated in a poly-D-Lysine coated 384-well plate with a volume of 25 µl per well which corresponds to about 7,500 cells per well. Each construct was transfected using Fugene 6.0 (Roche) according to the manufacturer’s protocol. Each plate contained cysteine-less and buffer controls. Essays were performed two days after transfection.

### FLIPR and SCAM Assay

Cells were washed three times with wash buffer (1× HANKS and 20 mM HEPES) using a M384 Atlas microplate washer (Titertek) and loaded with calcium-sensitive fluorophore Fluo-3 (Molecular Devices). After 1.5 hours Fluo-3 loaded plates were washed. MTSET stock solution was made by dissolving powder MTSET (Toronto Chemical) in ice-chilled distilled water and kept at −20°C up to three hours. 2×MTSET solutions (4 mM for TRPV1, 2 mM for TRPV3 screening) were made by dissolving MTSET stock in a wash buffer right before usage. 25 µl 2×MTSET solutions was added on 384 well plates and incubated for 5 min for TRPV1 and 10 min for TRPV3 at room temperature (20±2°C) or a water bath (42±2°C). As a negative control, buffer without MTSET was added. After MTSET incubation, 384-well plates were washed immediately three times. Fluorescence was measured by a FLIPR-Tetra plate reader (Molecular Devices).To measure temperature-activation, a custom-designed temperature-device [8] was used to raise temperature from 25°C to 45°C (for TRPV3) or 55°C (for TRPV1). Chemical activation was measured by adding various concentrations of capsaicin (for TRPV1) or 2-aminoethoxydiphenyl borate (2-APB) (for TRPV3).

### FLIPR Data Analysis and Statistics

Each representative graph was generated by averaging signals from 8 or more wells and error bars represent standard errors. The temperature in each well was calibrated by using the temperature-sensitive dye, [Ru(bpy)_3_]^2+^ (Sigma) and controlled by the temperature-device as previously described [8].

### MTSET Reactivity Assay

Reduced glutathione (GSH) and MTSET solution was prepared in distilled water. 20 µl 100 mM GSH was loaded on a 96-well fluorescence assay plate, and incubated on a temperature maintainer (Isotemp, Fisher Scientific). 20 µl of MTSET was added at various concentrations and mixed with GSH and incubated for 5 min. After incubation, the plate was removed from the temperature maintainer and 200 ul of 1 mg/ml OPA solution (ortho-phthalaldehyde solution, Sigma-Aldrich P0532) was added in each well and incubated for 1 min at room temperature. Fluorescence of the solution was measured in a fluorescence plate reader (Spectramax GeminiXPS, Molecular Devices); excitation at 340 nm, emission at 420 nm.

### Electrophysiology

HEK293 cells were co-transfected with EGFP and wild-type or mutant rat TRPV1 constructs (1∶5 ratio) in wells of 24-well plates using Fugene 6.0 (Roche). Cells were reseeded on 12 mm round glass coverslips (Warner Instruments) one day after transfection. Recordings were performed the following two days. Recording pipettes were pulled from micropipette glass (Sutter) and had 2–4 MΩ resistance. Whole-cell recordings were performed with pipette solution containing 150 mM NaCl, 3 mM MgCl_2_, 5 mM EGTA and 10 mM HEPES with adjusted pH 7.2 and bath solution containing 150 mM NaCl, 6 mM CsCl, 1.5 mM CaCl_2_, 1 mM MgCl_2_, 10 mM glucose and 10 mM HEPES with adjusted pH 7.4.

Voltage commands were made from an EPC9 (HEKA Instruments Inc) amplifier using the Pulse and PulseFit program, and the currents were filtered at 2.9 kHz and recorded at 10 kHz. Cells were continuously perfused with the bath solution through a Valve-Bank perfusion system (Automate Scientific). Temperature was controlled using a CL-100 temperature controller (Warner Instruments) and an SC-20 Solution In-Line Heater/Cooler (Harvard Apparatus) and measured with a thermistor placed in proximity to the cell. All experiments were performed within 1°C of the target temperature.

## Supporting Information

Figure S1 **Predicted location of screened residues.** Sequence alignment of pore domains of rat TRPV1 and mouse TRPV3. Screened residues are highlighted in gray.
(TIF)

Figure S2 **pH-dose responses of rTRPV1 Y653C.** Intracellular calcium levels of transiently transfected HEK293 cells in response to addition of buffer with low pH. (A) Fluorescence counts were normalized by the maximum value of fitting with Hill equations. (B) Same data as (A), but non-normalized. n>10 from two independent experiments. Error bars are mean ± s.e.
(TIF)

Figure S3 **Temperature-independent MTSET labeling on TRPV3 residues.** (A) Temperature as a function of time during FLIPR temperature-activation assay. (B) Representative examples of fluorescence responses upon temperature stimulation of TRPV3 I609C, K611C, S626C and L653C after incubation of MTSET at 20°C (blue) and 40°C (red). For both temperatures, a negative control (buffer) is shown as gray. n>7 wells. Error bars are mean ±2× s.e.
(TIF)
