# Supplementary figures and images for: Single Residues in the Outer Pore of TRPV1 and TRPV3 Have Temperature-Dependent Conformations
Source: PLoS One. 2013 Mar 26;8(3):e59593. doi: 10.1371/journal.pone.0059593 (PMC3608658; doi:10.1371/journal.pone.0059593)

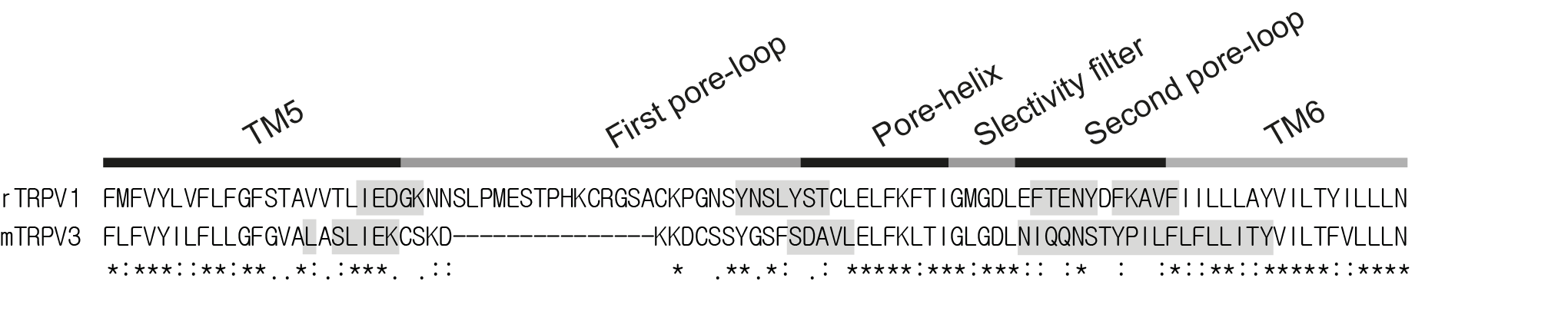

Supplement: Figure S1 — Predicted location of screened residues. Sequence alignment of pore domains of rat TRPV1 and mouse TRPV3. Screened residues are highlighted in gray. (TIF) [file pone.0059593.s001.tif]

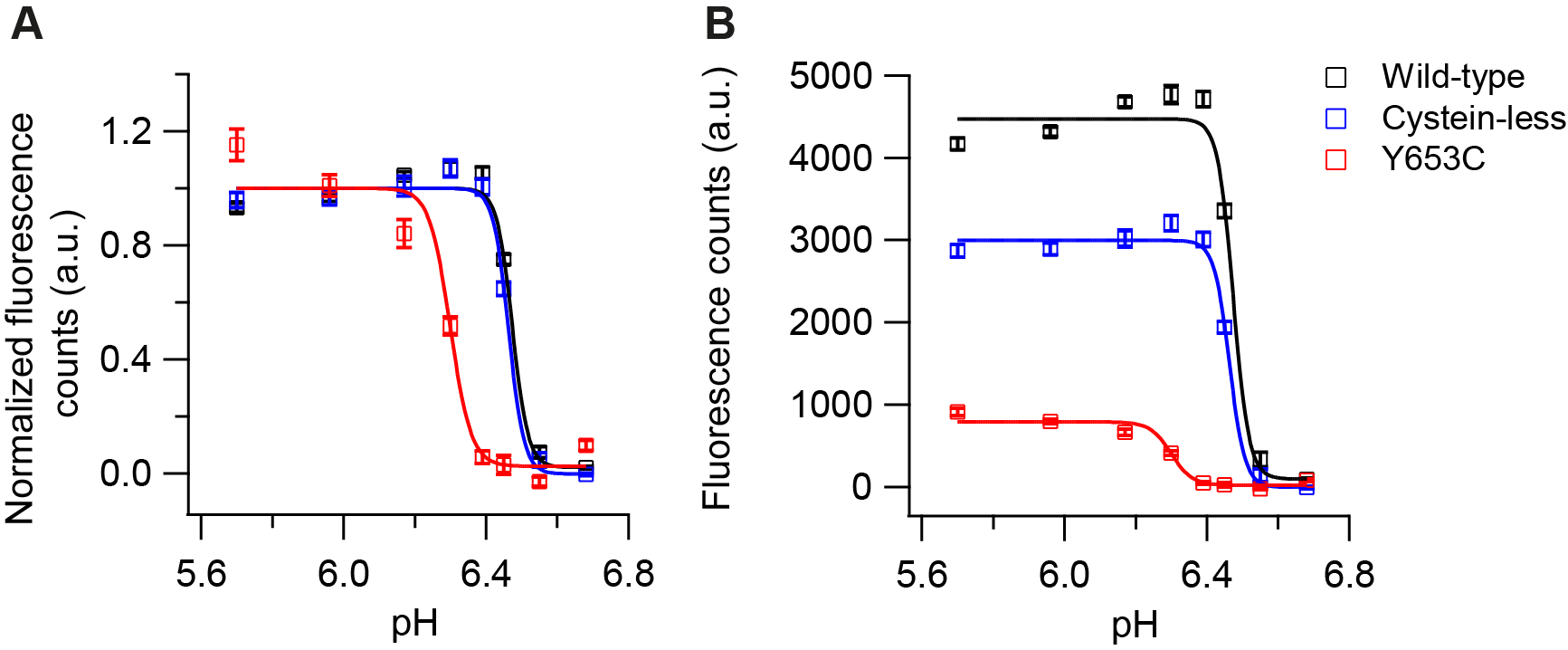

Supplement: Figure S2 — pH-dose responses of rTRPV1 Y653C. Intracellular calcium levels of transiently transfected HEK293 cells in response to addition of buffer with low pH. (A) Fluorescence counts were normalized by the maximum value of fitting with Hill equations. (B) Same data as (A), but non-normalized. n>10 from two independent experiments. Error bars are mean ± s.e. (TIF) [file pone.0059593.s002.tif]

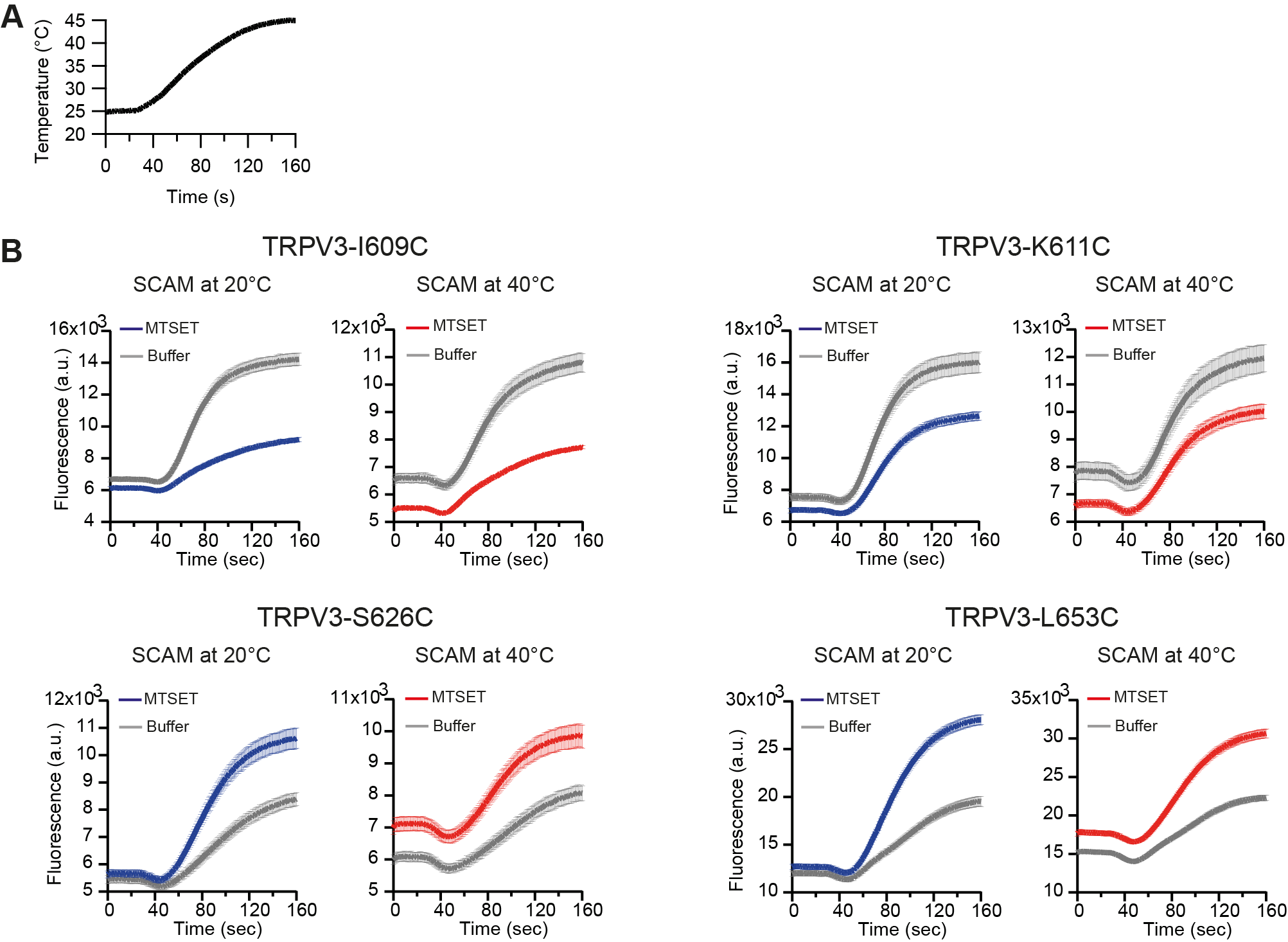

Supplement: Figure S3 — Temperature-independent MTSET labeling on TRPV3 residues. (A) Temperature as a function of time during FLIPR temperature-activation assay. (B) Representative examples of fluorescence responses upon temperature stimulation of TRPV3 I609C, K611C, S626C and L653C after incubation of MTSET at 20°C (blue) and 40°C (red). For both temperatures, a negative control (buffer) is shown as gray. n>7 wells. Error bars are mean ±2× s.e. (TIF) [file pone.0059593.s003.tif]
